# Salsalate Prevents β-Cell Dedifferentiation in OLETF Rats with Type 2 Diabetes through Notch1 Pathway

**DOI:** 10.14336/AD.2018.1221

**Published:** 2019-08-01

**Authors:** Fei Han, Xiaochen Li, Juhong Yang, Haiyi Liu, Yi Zhang, Xiaoyun Yang, Shaohua Yang, Bai Chang, Liming Chen, Baocheng Chang

**Affiliations:** ^1^NHC Key Laboratory of Hormones and Development (Tianjin Medical University), Tianjin Key Laboratory of Metabolic Diseases, Tianjin Medical University Metabolic Diseases Hospital & Tianjin Institute of Endocrinology, Tianjin, China; ^2^Tianjin Medical University General Hospital, Tianjin Medical University, Tianjin, China

**Keywords:** Notch1 pathway, Salsalate, Type 2 diabetes, β-cell dedifferentiation

## Abstract

A strategic approach is urgently needed to curb the growing global epidemic of diabetes. In this study, we investigated the effects and mechanisms of salsalate (SAL), an anti-inflammatory drug with anti-diabetic properties, assessing its potential to prevent diabetes in Otsuka Long-Evans Tokushima Fatty rats (OLETF). All animals in our placebo group developed diabetes, whereas none in the SAL test group did so, and only 25% of SAL-treated rats displayed impaired glucose tolerance (IGT). SAL lowered levels of glucagon and raised levels of insulin in plasma, while improving both insulin sensitivity and β-cell function. The protective effect of SAL is likely due to diminished β-cell dedifferentiation, manifested as relative declines in Neurogenin 3^+^/insulin^-^ cells and synaptophysin^+^/islet hormone^-^ cells and increased expression of β-cell-specific transcription factor Foxo1. Both Notch1-siRNA and N-[N-(3,5-difluorophenacetyl)-1-alanyl]-S-phenylglycine t-butyl ester (DAPT; an indirect inhibitor of the Notch1 pathway) were shown to prevent β-cell dedifferentiation. Similar to DAPT, SAL effectively reduced β-cell dedifferentiation, significantly suppressing Notch1 pathway activation in INS-1 cells. The inhibitory role of SAL in β-cell dedifferentiation may thus be attributable to Notch1 pathway suppression.

Type 2 diabetes (T2D) is a major global health burden associated with high morbidity and considerable mortality. T2D has been linked to progressive β-cell failure stemming from the reduced insulin secretion and depleted β-cell reserves [1]. The latter is initially triggered as a compensatory response to peripheral insulin resistance, although eventually there is loss of the mature β-cell phenotype without mandatory β-cell death [2, 3]. This phenotypic loss, sometimes referred to as dedifferentiation, may result from exposure to high levels of glucose, lipids, and inflammatory cytokines [4].

Notch signaling serves redundantly during organ and tissue development to effect lineage segregation of progenitor cells. It is essential to maintain a pool of proliferating endocrine progenitor cells and confer timely cell lineage specification during exocrine and endocrine cell differentiation [5]. In the developing pancreas, important cell-fate decisions are regulated by Notch receptors, which forward signals to the Hairy and Enhancer of Split 1 (Hes1) transcription regulator. Evidence has shown that the Notch-Hes1 signaling pathway participates in β-cell dedifferentiation [6, 7].

The hypoglycemic effect of salicylates has been known for some time, demonstrated as early as 1876. Salicylates exist in two distinct forms: aspirin (ASA), the acetylated prototypic agent, and salsalate (SAL), the nonacetylated drug [8]. Aspirin inhibits cyclooxygenase enzymes, which leads to alterations in bleeding time and platelet aggregation and may even cause gastric bleeding at higher dosages [9]. SAL has typically been used to treat inflammatory conditions and has fewer side effects than aspirin [10]. In particular, SAL carries no heightened risk of gastrointestinal bleeding. It is therefore relatively safe for long-term clinical use and is thus considered a promising anti-diabetic medication [11].

In a study conducted at the Joslin Diabetes Center, patients with T2D were randomly assigned to a 14-week course of placebo or SAL, administered in conjunction with their regular treatments. The subsequent findings indicated that SAL lowered blood glucose, circulating triglycerides, and free fatty acid concentrations and raised adiponectin concentrations [12]. Although there is other evidence that SAL acts to improve weight gain, lipid metabolism [13, 14], and insulin sensitivity [15], the related mechanisms are still largely unknown.

In this study, we assessed the anti-diabetic potential of SAL in Otsuka Long-Evans Tokushima Fatty (OLETF) rats and explored its likely mechanism of action, focusing on modulation of β-cell dedifferentiation via the Notch1 pathway.

## MATERIALS AND METHODS

### Animals

All animal experiments complied with the rules of the Experimental Animal Care and Use Center at Tianjin Medical University. The protocol was approved by the Experimental Animal Ethical Committee of Tianjin Medical University. Four-week-old male OLETF rats (n=40) and Long-Evans Tokushima Otsuka (LETO) rats (n=20) were purchased (Otsuka Pharmaceutical, Tokushima, Japan). The OLETF rats is a genetic model of late-onset spontaneous obese-related T2D, demonstrating insulin resistance and hyperinsulinemia as well as with hyperglycemia [16]. The aging rats eventually develop hypoinsulinemia due to deterioration in islet β cells [17, 18].

In our experiment, each animal was housed individually, with free access to food and water. They were maintained in a controlled room at 22°C, with a 12-h day/night cycle. After adaption, the OLETF rats were randomly divided into diabetic (n=20) and SAL-treated (n=20) group. This took place at 24 weeks of age, the point at which insulin resistance and hyperinsulinemia are anticipated [19-21]. LETO rats served as normal controls (n=20). All groups were fed* ad libitum*, mixing 250 mg/kg·d^-1^ SAL (LKT Laboratories, St Paul, MN, USA) in the food of SAL-treated rats [22]. Oral glucose tolerance tests (OGTTs) were performed and fasting insulin levels were monitored every 16 weeks. After treatment for 32 weeks, ratios of diabetes and impaired glucose tolerance (IGT) in the various groups were calculated, based on OGTT results.

### Cell Culture

Rat INS-1 cells (American Type Culture Collection, Manassas, VA, USA) were cultured in RPMI 1640 medium containing 10% fetal bovine serum (Gibco, Gaithersburg, MD, USA) and 50 μM β-mercaptoethanol (Gibco) at 37°C in 5% CO_2_. The cells were then stimulated for 48 h by normal (5.5 mM) or high (25 mM) concentrations of D-glucose (Sigma-Aldrich, St Louis, MO, USA) in RPMI 1640 medium plus 0.1% BSA. D-mannitol (25 mM; Sigma-Aldrich) served as osmolality control. SAL (LKT Laboratories, USA) was reconstituted in 0.01% dimethyl sulfoxide and freshly diluted with culture medium before use. A 1-μM aliquot of N-[N-(3,5-difluorophenacetyl)-1-alanyl]-S-phenylglycine t-butyl ester (DAPT; MedChem Express, Monmouth Junction, NJ, USA) was also dispensed to cultured cells. Notch1 siRNA was transfected using a commercial kit (riboFECT CP Transfection Kit; RiboBio, Guangzhou, China) as instructed by manufacturer.

### OGTT

After fasting for 15 hours and undergoing glucose (2 g/kg body weight) gavage, blood glucose levels of rats were measured at 0, 30, 60, and 120 min via automated analyzer (Roche Diagnostics, Mannheim, Germany) to calculate the following:

AUCg = (G_0_+G_120_)/4+(G_30_+G_60_)/4+(G_60_+G_120_)/2

HOMA-β = 20×FINS/(FBG-3.5)

HOMA-IR = FBG× FINS/22.5.

AUCg: area under the curve for glucose; G: blood glucose level (0 min [G_0_], 30 min [G_30_], 60 min [G_60_], and 120 min [G_120_]); FINS: fasting plasma insulin; FBG: fasting blood glucose level

### Diagnostic criteria

Rats were considered diabetic if they showed a peak blood glucose level ≥16.7 mmol/L and a G_120_ (120-min) blood glucose level ≥11.1 mmol/L, and levels beyond one of these thresholds signaled IGT [23].

### Blood Biochemical Markers

Blood was collected from inner canthal orbital vein after overnight fasting. Concentrations of plasma insulin (GBD, San Diego, CA, USA) and glucagon (Solarbio Science and Technology, Beijing, China) were determined by ELISA.

**Figure 1.**
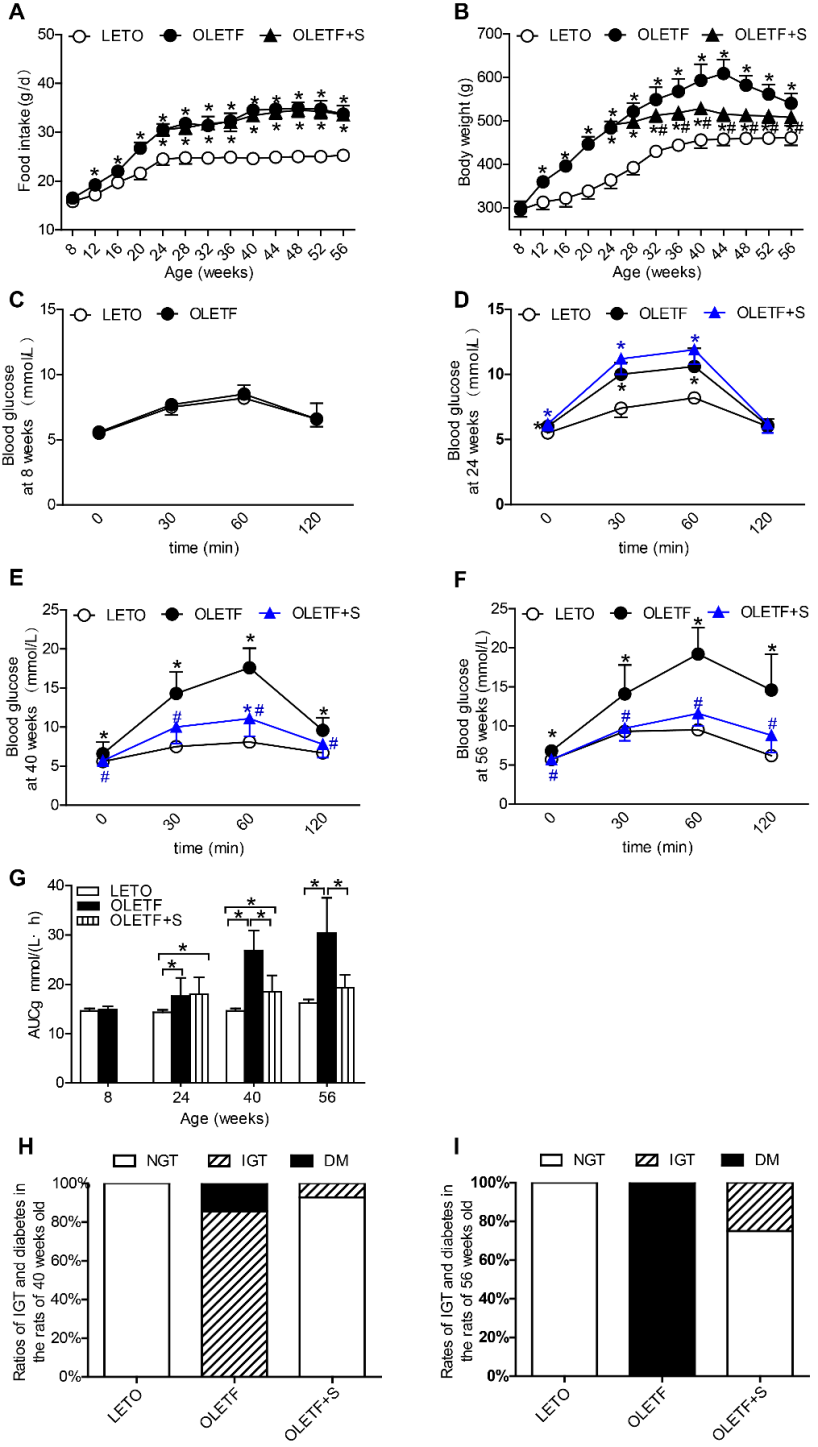
SAL prevents onset of diabetes in OLETF rats (**A**) Serial changes in food intake; (**B**) Serial changes in body weight; (**C-F**) OGTT results in rats at ages 8 (**C**), 24 (**D**), 40 (**E**) and 56 (**F**) weeks. Data are reported as mean ± SEM. *P < 0.05 vs. LETO rats, #*P* < 0.05 vs. OLETF rats; (**G**) AUCg values determined by OGTT. Data are reported as mean ± SEM. *P < 0.05; H and I: Ratios of IGT and diabetes in rats at ages 40 (H) and 56 (**I**) weeks.

**Figure 2.**
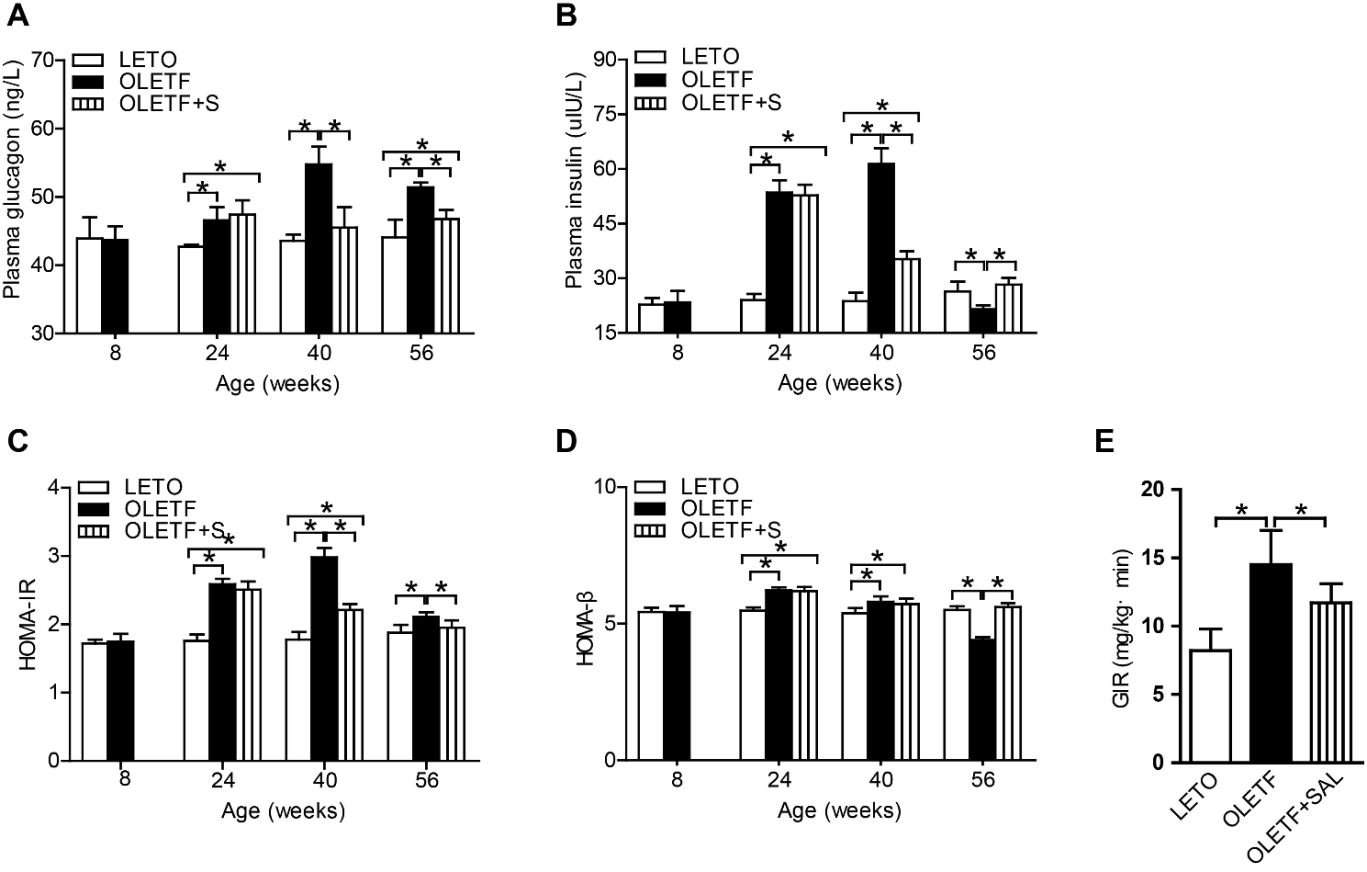
SAL improves islet-cell function and insulin resistance in OLETF rats Fasting plasma glucagon (**A**) and insulin (**B**) concentrations; (**C**) HOMA-β values in rats. (**D**) HOMA-IR values in rats. (**E**) GIRs in rats. Data are reported as mean ± SEM. *P < 0.05.

### Tissue collection and processing

Pancreatic organs were dissected and rapidly separated into two parts: one placed in liquid nitrogen and the other fixed in 4% paraformaldehyde.

### Islet isolation

Rats were anesthetized by intraperitoneal injection of 10% chloral hydrate (0.3 ml/kg). In each instance, we cannulated the bile duct and perfused Hank’s solution containing collagenase (Roche Diagnostics), subsequently removing the pancreas for digestion in a 37ºC water bath. After shaking in a centrifuge tube and washing in cold Hank’s solution, islets were handpicked under a stereomicroscope for storage at -80°C.

### Morphometric measurements

Hematoxylin and eosin staining enabled general assessment of islet morphology. The ultrastructure of islet cells was captured by transmission electron microscopy (EM).

### Immunofluorescence and densitometry

Formalin-fixed pancreatic samples were embedded into paraffin blocks. Representative sections (5 µm) were incubated overnight with the following primary antibodies: anti-glucagon (1:200; Cell Signaling Technology, Danvers, MA, USA); anti-insulin (1:400; Cell Signaling Technology or Proteintech Group, Rosemont, IL, USA); anti-Neurogenin 3 (Ngn3) (1:50; Santa Cruz Biotechnology, Dallas, TX, USA); anti-synaptophysin (Syn) (1:50; Cell Signaling Technology); anti-somatostatin (Ssn) (1:50; Proteintech Group); anti-Forkhead box-containing protein O1 (Foxo1) (1:100; Cell Signaling Technology); or anti-Notch1 (1:50; Cell Signaling Technology). After incubation with ?uorescein-labeled anti-rabbit and anti-mouse secondary antibodies, the slides were mounted and imaged via confocal microscope. For quantitative analytics, we scored at least three random sections per pancreas and five random islets per section.

**Figure 3.**
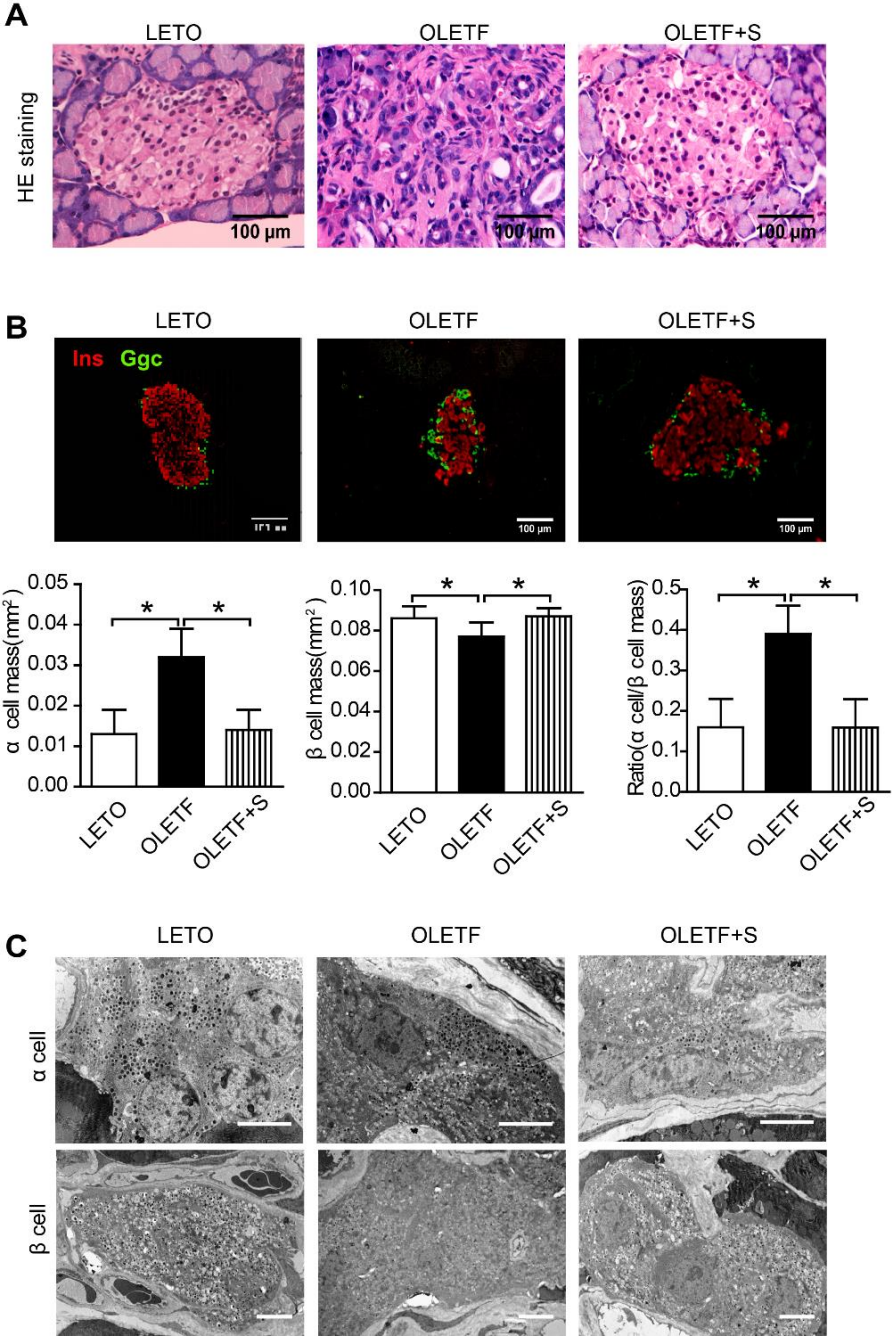
SAL restores morphology and architecture of islets in OLETF rats (**A**) H&E staining of islets in rats. The scale bar represents 100 μm. (**B**) Immunofluorescence images of islets stained for insulin (Ins) (red) and glucagon (Ggc) (green). The scale bar represents 100 μm. Data are reported as mean ± SEM. *P < 0.05. (**C**) EM details of α and β cells in various groups. The scale bar represents 1 μm.

### Western blot analysis

Once total pancreatic protein isolates were obtained and the concentrations tested, the protein extracts (40 μg/lane) were separated using SDS-PAGE, then transferred to polyvinylidene ?uoride membranes (MilliporeSigma, Burlington, MA, USA) for overnight incubation with mouse anti-β-actin (1:8000; Sungene Biotech, Tianjin, China), rabbit anti-Notch1 (1:5000; Cell Signaling Technology), rabbit anti-Notch intracellular domain (NICD, 1:5000; Cell Signaling Technology), rabbit anti-Hes1 (1:500; Abcam, Cambridge, UK), rabbit anti-Foxo1 (1:500; Cell Signaling Technology) or rabbit anti-Ngn3 (1:50; Santa Cruz Biotechnology) antibodies. After washing, an appropriate secondary antibody was applied for detection. Proteins were visualized by enhanced chemiluminescence (ECL; Advansta, San Jose, CA, USA), using ImageJ open source software to analyze band intensities.

### Hyperinsulinemic-euglycemic clamp

A hyperinsulinemic-euglycemic clamp was used to test the insulin resistance of 56-week-old rats. After anesthetization, two catheters were inserted (one in each femoral vein) for infusion of glucose or insulin (Novolin R; Novo Nordisk, Bagsværd, Denmark) by injection pump (Alaris GH; BD, Franklin Lakes, NJ, USA). A third catheter was inserted into the carotid artery for sampling of blood. Insulin was infused at a constant rate of 20 mU/(kg·min), and arterial blood glucose concentration was maintained at basal fasting level by infusing 25% glucose at variable rates. Under hyperinsulinemic conditions, the steady glucose infusion rate (GIR) required to maintain euglycemia (usually calculated between 60-120?min) is a standard measure of whole-body insulin sensitivity.

### Statistical analysis

Standard software (SPSS v19.0; IBM, Armonk, NY, USA) was engaged, subjecting all data to one-way analysis of variance (ANOVA) or a least significant difference (LSD) test and expressing values as mean ± SEM. Statistical significance was set at *p*<0.05.

## RESULTS

### SAL prevented onset of diabetes in OLETF rats

Body weights of OLETF rats declined significantly after SAL intake with unaltered food intake (Fig. 1A, B). Relative to LETO rats, blood glucose levels of untreated OLETF rats were significantly higher at baseline and within 1 hour (30 and 60 min) of glucose loading at ages 24, 40, and 56 weeks and after 120 min at ages 40 and 56 weeks. However, corresponding blood glucose levels were significantly lower in rats given SAL (Fig. 1C-F). The resultant AUCg value underscores the SAL-related hypoglycemic effect (Fig. 1G). We calculated the ratio of rats with abnormal glucose tolerance to rats with normal glucose tolerance based on OGTT results. Overall, 85.7% of untreated OLETF rats developed IGT at 40 weeks of age, the remainder developing developed diabetes. At the same time, only 7.1% of the treated group developed IGT, with all other displaying normal glucose tolerance (Fig. 1H). All untreated OLETF rats developed diabetes at 56 weeks old, but only 25% of SAL-treated rats progressed to IGT, and none advanced to overt diabetes (Fig. 1I).

### SAL improved both islet-cell function and insulin resistance in OLETF rats

Fasting plasma glucagon concentration, which was elevated in OLETF rats at 24 weeks and increased further at 40 and 56 weeks, was lowered significantly by SAL (Fig. 2A). Fasting plasma insulin concentration was significantly increased in OLETF rats at 24 and 40 weeks and markedly declined at 56 weeks. SAL intervention alleviated hyperinsulinemia in OLETF rats (Fig. 2B). HOMA-β values were calculated to measure β-cell function, which was significantly reduced in diabetic OLETF rats but was largely preserved in the SAL-treated group (Fig. 2C). HOMA-IR values were calculated to evaluate insulin resistance. Substantial insulin resistance was observed in the early phase of OLETF rats and persisted throughout the course of testing, but insulin resistance was significantly reduced by SAL (Fig. 2D). Insulin sensitivity, represented by GIR (8.2±1.6 mg/kg·min^-1^) in diabetic OLETF rats, was lower than that of LETO rats (14.5±2.5 mg/kg·min^-1^) and was restored by SAL treatment (11.7±1.4 mg/kg·min^-1^) (Fig. 2E).

### SAL treatment restored islet morphology and islet architecture in OLETF rats

Islets of diabetic OLETF (vs LETO) rats were in disarray, trailing into adjacent exocrine tissue and permeated by adenoid elements, whereas in SAL-treated rats, islet morphology remained essentially intact (Fig. 3A).

Islet architecture in LETO rats was typical, characterized by placed α cells and central β-cell aggregates. In islets of OLETF rats, peripheral α cells impinged on β-cell clusters, assuming predominance and suggesting conversion of β cells to α-like glucagon-producing cells [24]. Indeed, the α-cell mass of OLETF rats was significantly increased compared with that of LETO rats, and the β-cell mass of the OLETF rats was diminished, significantly altering the α-to-β cell mass ratio in diabetic OLETF rats (Fig. 3B). These findings are thus indicative of β-cell dedifferentiation in OLETF rats. On the other hand, SAL-treated rats showed a significant decline in α-cell mass and a lower α-to-β cell mass ratio (Fig. 3B).

Ultrastructural details of islet cells were aligned with above results. Compared with the status of LETO rats α cells, the more numerous secretory granules of α cells seen in OLETF rats were otherwise reduced by SAL treatment, and the scant secretory granules found in most β cells of diabetic OLETF rats were thereby restored (Fig. 3C).

### SAL prevented β-cell dedifferentiation in OLETF rats

**Figure 4.**
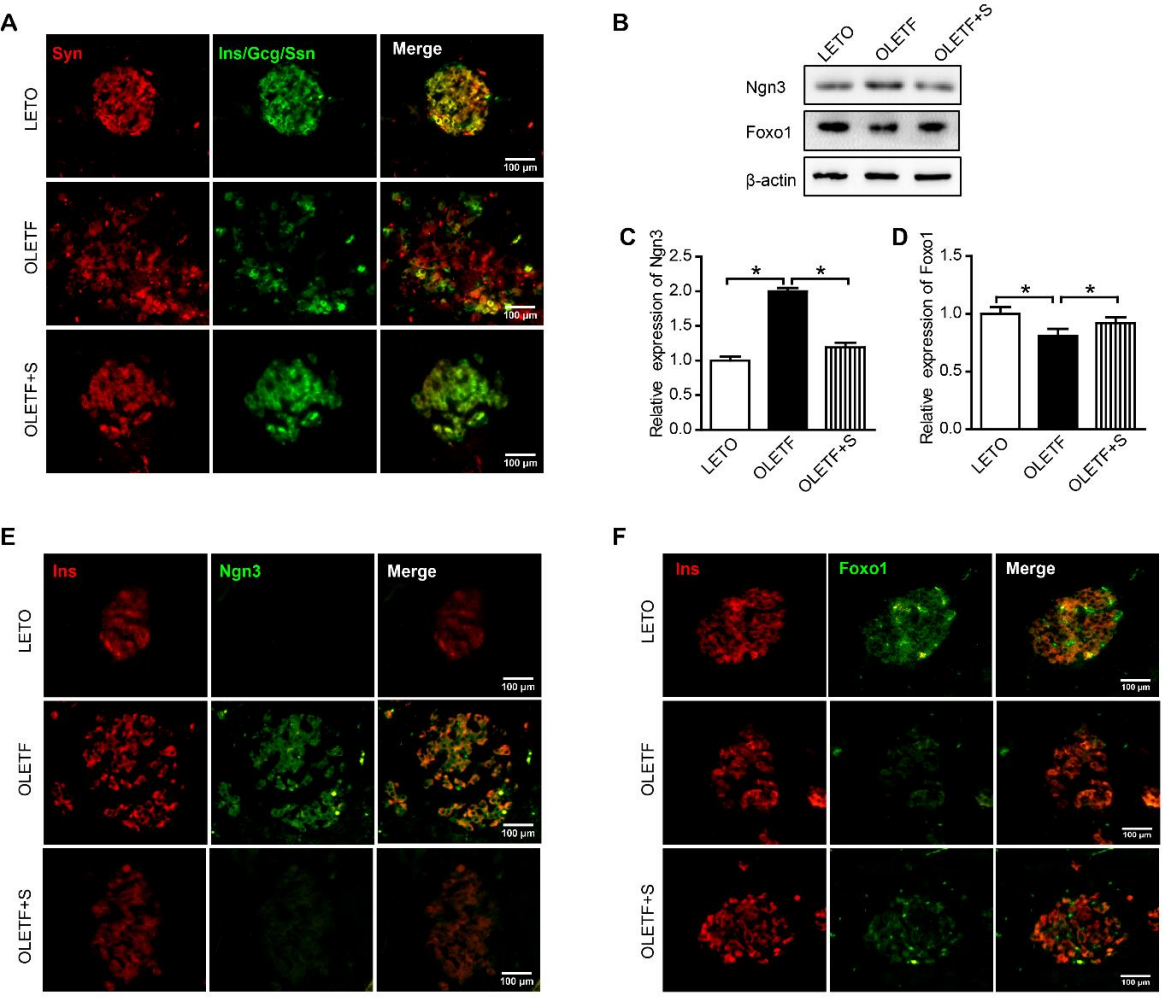
SAL prevents β-cell dedifferentiation in OLETF rats (**A**) Immunofluorescence images of rat pancreatic tissue stained for Syn (red), Ins, Ggc, and Ssn (green); (**B**) Expression levels of Ngn3 and Foxo1 proteins in isolated islet tissue samples; (**C**) Quantified Ngn3 protein expression levels; (**D**) Quantified Foxo1 protein expression levels; (**E**) Immunofluorescence images of rat pancreatic tissue stained for Ins (red) and Ngn3 (green). (**F**) Immunofluorescence images of rat pancreatic tissue stained for Ins (red) and Foxo1 (green). The scale bar represents 100 μm. Data are reported as mean ± SEM. *P < 0.05.

By definition, the dedifferentiated β cell is Syn positive cell and shows no immune reactivity to the five pancreatic hormones: insulin, glucagon, pancreatic polypeptide (PP), Ssn, and ghrelin. We tested for hormone positivity, using antibodies to insulin, glucagon, and Ssn and general endocrine features, using antibodies to Syn [25-27]. Compared with LETO rats, OLETF rats showed a significant increase in the percentage of Syn^+^ hormone^-^ cells, indicating a loss of hormone expression (i.e., dedifferentiation) in some islet endocrine cells. This was not observed in islets of SAL-treated rats (Fig. 4A).

The concept of β-cell dedifferentiation, marked by loss of β-cell insulin expression and increased expression of Ngn3 (a marker of islet progenitor cells [28]), has been corroborated in animal models of diabetes [25, 29]. We used western blot to test for Ngn3 protein expression in pancreatic islets. Ngn3 expression proved significantly greater in OLETF (vs LETO) rats but was prevented by SAL treatment (Fig. 4B, C). Pancreatic tissue sections were also immunostained for insulin (red) and Ngn3 (green), revealing high numbers of dedifferentiated cells in islets of OLETF rats. Importantly, most Ngn3^+^ cells were insulin^-^ as well. SAL treatment significantly improved these alterations in OLETF rats (Fig. 4E).

Dedifferentiation of β cells in the context of diabetes has been shown to occur* in vivo* through genetic disruption of the key transcription factor Foxo1 [25]. In this study, a significant reduction in expression of Foxo1 shown by OLETF rats was improved through SAL treatment (Fig. 4B, D). As depicted in immunofluorescence images, diminished Foxo1 levels in OLETF rats paralleled the loss of insulin immunoreactivity, both of which were restored by SAL treatment (Fig. 4F).

**Figure 5.**
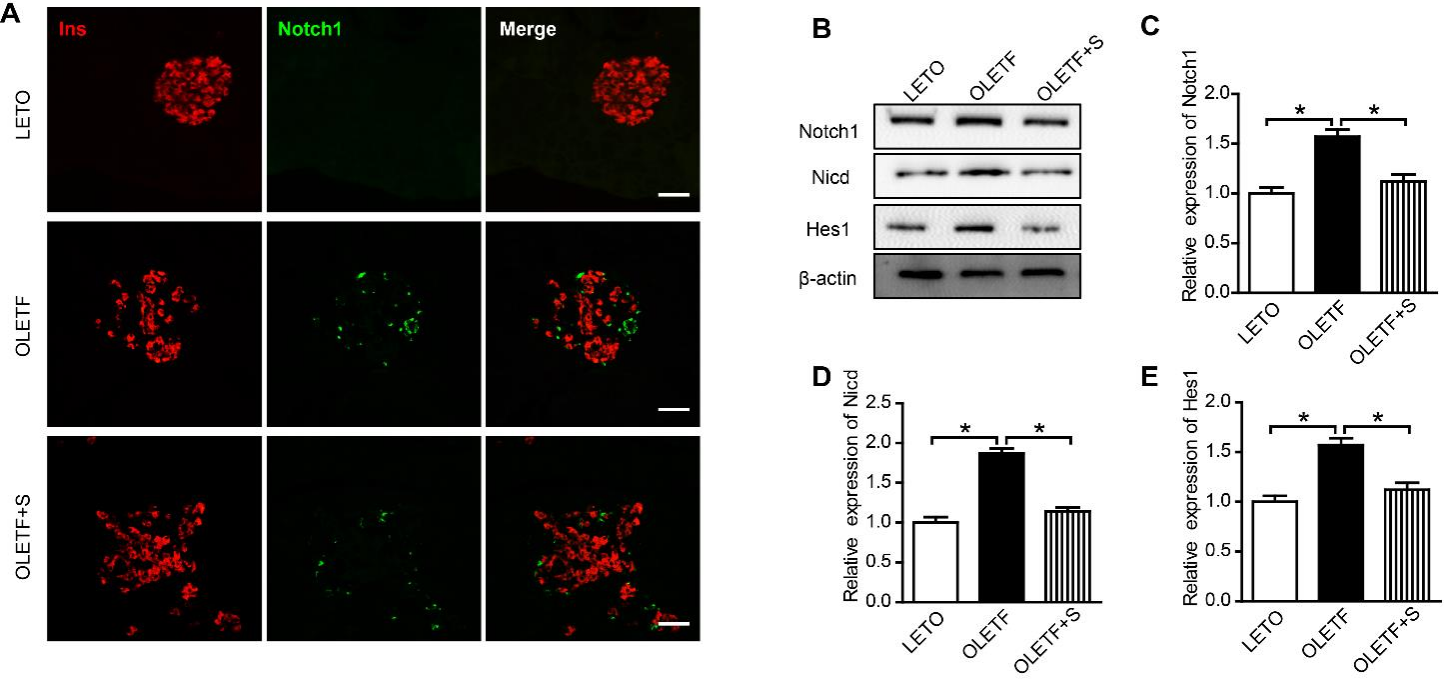
SAL inhibits Notch1 pathway in islets of OLETF rats (**A**) Immunofluorescence images of islets stained for Ins (red) and Notch1 (green); (**B**) Western blot images of Notch1 pathway-related protein expression; (**C**) Western blot results of the expression of Notch1. (**D**) Western blot results of the expression of Nicd. (**E**) Western blot results of the expression of Hes1. The scale bar represents 100 μm. Data are reported as mean ± SEM. *P < 0.05.

### SAL inhibited Notch1 pathway in islets of OLETF rats

At present, there is evidence that Notch-Hes1 signaling pathway may participate in β-cell dedifferentiation [6, 7]. Immunofluorescence staining of Notch1 showed its significant activation in islets of OLETF rats, although SAL treatment diminished Notch1 activation significantly (Fig. 5A). We further examined the effect of SAL on the Notch1 pathway in pancreatic islets by western blot (Fig. 5B-E). Notch1 is a plasma membrane receptor that is proteolytically cleaved upon binding with its ligands, releasing the Nicd. Nuclear NICD translocation then activates the specific target gene, Hes1. In our study, the Notch1 pathway was activated in islets of OLETF rats but was suppressed by SAL treatment (Fig. 5B-E).

### Inhibitory effect of SAL in β-cell dedifferentiation linked to suppression of Notch1 pathway

To further clarify the role of the Notch1 pathway in β-cell dedifferentiation imposed by T2D, Notch1 siRNA was transfected into INS-1 cells cultured under high-glucose conditions.

**Figure 6.**
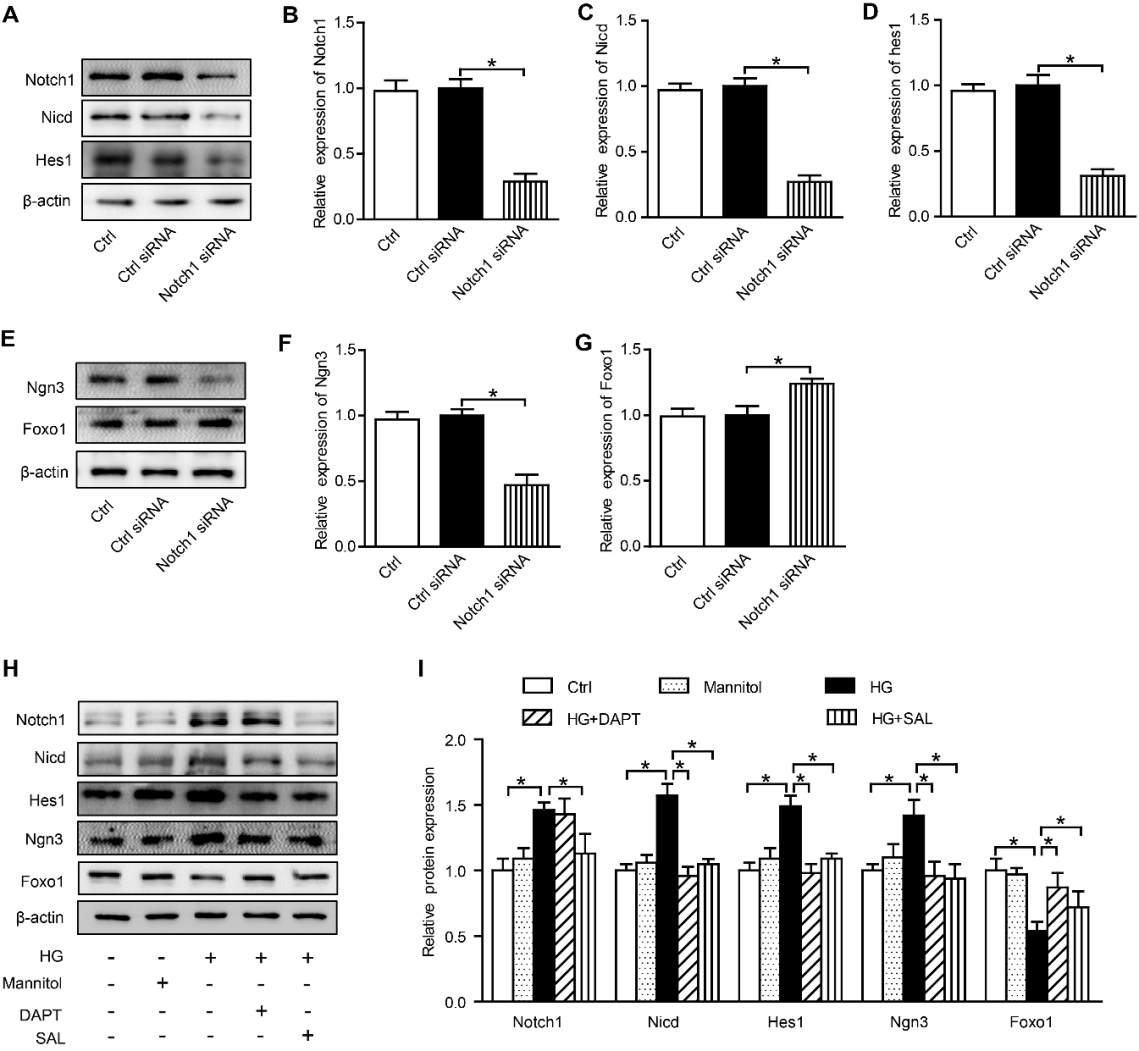
SAL inhibition of β-cell dedifferentiation attributable to suppression of Notch1 pathway (**A**) Western blot images of Notch pathway-related protein expression in cells transfected by Notch1-siRNA; (**B-D**) Quantification of results in A; (**E**) Western blot images of Ngn3 and Foxo1 expression levels in cells transfected with Notch1-siRNA. (**F-**G) Quantification of results in E; H: Western blot images of protein expression in INS-1 cells treated for 48 h. I: Quantification of results in H. Data are reported as mean ± SEM. *P < 0.05.

As our data show, protein levels of Notch1, Nicd, and Hes1 declined significantly after transfection with Notch1 siRNA, so this worked well as a deterrent (Fig. 6A-D). Furthermore, Ngn3 protein expression was reduced (Fig. 6E, F), and Foxo1 protein expression increased (Fig. 6E, G) owing to knockdown of Notch1. DAPT is an agent that prevents Nicd release from Notch1 and is recognized as an indirect inhibitor of the Notch1 pathway [30]. Use of DAPT also inhibited the expression of Notch1 and promoted the expression of Foxo1, as did Notch1 siRNA (Fig. 6H, I). Similar to DAPT, SAL inhibited activation of the entire Notch1 pathway in high glucose-treated INS-1 cells, and inhibition of the Notch1 signaling pathway by SAL decreased expression of Ngn3 and increased Foxo1 expression. Our findings imply that perturbation in Notch1 signaling may help prevent β-cell dedifferentiation and that the inhibitory effects of SAL in β-cell dedifferentiation may be attributable to its suppression of the Notch1 pathway.

## DISCUSSION

The present study was designed to assess the anti-diabetic effects of SAL in OLETF rats and examine the related mechanisms. OLETF rats are known for their genetic predisposition to late-onset spontaneous development of obese-related T2D, showing early hyperinsulinemia and insulin resistance early and progressing from IGT to overt diabetes over time as islet β cells deteriorate [17, 18, 31]. These clinical and pathological features closely resemble those seen in human with T2D [32]. Herein, all untreated OLETF rats developed diabetes by 56 weeks of age, whereas diabetes was absent in SAL-treated counterparts, and only 25% showed demonstrable IGT.

Insulin resistance is typical of T2D, accompanied by changes in lipid metabolism, oxidative stress, inflammation, etc. Our findings show that SAL improves insulin resistance in OLETF rats, corroborating data in previous reports [15]. SAL also conferred functional improvement in islet β cells of OLETF rats. Progressive β-cell failure, stemming from insulin resistance, is a key determinant of outcomes in patients with T2D. Although insulin resistance remains relatively stable over time, β-cell function in this setting is destined for rapid, steady decline [33, 34]. Our data suggest that the protective effects of SAL in diabetes-prone OLETF rats are related to enhanced β-cell function. The amelioration of the HOMA-β values and restitution of islet morphology/architecture that we observed serve as proof.

Similarly, SAL decreased levels of glucagon and increased levels of insulin in plasma. Outcomes of dual insulin and glucagon immunostaining and EM ultrastructural details of β cells likewise support the anti-diabetic properties of SAL in this animal model. EM evidence indicates that degranulation occurs as rats develop diabetes. Thus, the α-to-β cell ratio is significantly increased in diabetic OLETF rats, due to fewer insulin-containing and more glucagon-containing cells. Such pathologic transformations are shared by diabetic humans [35, 36]. Furthermore, the typical cellular arrangement of islets (i.e., α cells at periphery, β cells aggregated at center) in pancreatic samples of OLETF rats is progressively lost as diabetes ensues, with α cells impinging on β-cell clusters. It is therefore quite feasible that β cells morph into other islet cell types, perhaps α-like glucagon-producing variants [24, 25], accounting for the hyperglucagonemia and hypoinsulinemia of OLETF rats.

Another issue is that adjacent exocrine and adenoid tissues had encroached on islets in areas ordinarily populated by β cells, suggesting that abnormal differentiation had occurred. Recently, an alternative mechanism for diabetic failure seems to have surfaced. Talchai *et al.* have reported the dedifferentiation of β cells into endocrine progenitor-like cells during stress-induced hyperglycemia [25], and a similar phenomenon has been observed *in vitro* [37], in diabetic mice [29], and in human with T2D [26]. In our investigation as well, β-cell dedifferentiation was verifiable in diabetic OLETF rats and was partly reversed by SAL.

Expression of Ngn3, a marker of islet progenitor cells [28], was clearly enhanced in OLETF rats with diabetes and was diminished during SAL treatment. Moreover, most Ngn3^+^ cells in islets of diabetic rats were also negative for insulin, indicating that a large portion of the mature insulin-containing β cells had been replaced. Such Ngn3^+^ and insulin^-^ cells are thought to arise from dedifferentiation of mature β cells [29]. Furthermore, dual staining for Syn and islet hormones revealed that although most of such cells retained endocrine properties under diabetic conditions, they lost hormonal immunoreactivity, again supporting the concept of β-cell dedifferentiation. Finally, we also examined the effect of SAL on the β cell-specific transcription factor Foxo1 [38]. Foxo1 is a major determinant of cell fate in β cells. A defect in Foxo1 expression demonstrated by OLETF rats contributes to functional failure of β cells and provides even further evidence β-cell dedifferentiation. As already mentioned, one prior report has cited reversion to progenitor-like endocrine cells during stress-induced hyperglycemia in islets that lack Foxo1 expression [25]. Thus, restoring Foxo1 expression to deter β-cell dedifferentiation is seemingly an important therapeutic benefit of SAL.

Ultimately, the underlying molecular mechanism by which SAL treatment prevents β cell dedifferentiation is still unclear. Notch signaling is redundantly used during the development of organs and tissues to determine the lineage segregation of progenitor cells. In the developing pancreas, important cell-fate decisions are regulated by Notch receptors, which signal through the Hairy and Enhancer of Split 1 (Hes1) transcription regulator. Evidence has shown that the Notch-Hes1 signaling pathway participates in β-cell dedifferentiation [6, 7]. We knocked down Notch1 expression in high glucose-treated INS-1 cells using siRNA, which prevented β-cell dedifferentiation, and exposure to DAPT (a Notch pathway inhibitor) produced the same result. Importantly, SAL functioned similarly to DAPT in this regard, significantly suppressing Notch1 pathway activation in INS-1 cells. These results imply that the inhibitory effects of SAL in terms of β-cell dedifferentiation may be attributable to Notch1 pathway inhibition.

In conclusion, findings of the present study indicate that β-cell dysfunction and insulin resistance jointly contribute to progression of diabetes in OLETF rats. SAL administration clearly protected the OLETF rats we studied by effecting improvements on both counts. SAL may act to prevent β-cell dedifferentiation (and thus avert diabetes) by inhibiting the Notch1 pathway. Although this effort has broadened our awareness of SAL and its role in preventing T2D, other uses of this safe and inexpensive drug must await further research.
